# Cytokine profiles of plasma extracellular vesicles as progression biomarkers in Parkinson’s disease

**DOI:** 10.18632/aging.204575

**Published:** 2023-03-09

**Authors:** Lung Chan, Chen-Chih Chung, Ruan-Ching Yu, Chien-Tai Hong

**Affiliations:** 1 Department of Neurology, Shuang Ho Hospital, Taipei Medical University, Taipei, Taiwan; 2 Department of Neurology, School of Medicine, College of Medicine, Taipei Medical University, Taipei, Taiwan; 3 Taipei Neuroscience Institute, Taipei Medical University, Taipei, Taiwan; 4 Division of Psychiatry, University College London, London, UK

**Keywords:** extracellular vesicles, Parkinson’s disease, inflammation, cytokine

## Abstract

Background: Inflammation contributes substantially to the pathogenesis of Parkinson’s disease (PD). Plasma extracellular vesicle (EV)-derived cytokines are emerging biomarkers of inflammation. We conducted a longitudinal study of the plasma EV-derived cytokine profiles of people with PD (PwP).

Methods: A total of 101 people with mild to moderate PD and 45 healthy controls (HCs) were recruited, and they completed motor assessments (Unified Parkinson Disease Rating Scale [UPDRS]) and cognitive tests at baseline and 1-year follow-up. We isolated the participants’ plasma EVs and analyzed their levels of cytokines, including interleukin (IL)-1β, IL-6, IL-10, tumor necrosis factor (TNF)-α, and transforming growth factor (TGF)-β.

Results: We noted no significant changes in the plasma EV-derived cytokine profiles of the PwPs and HCs between baseline and the 1-year follow-up. Among the PwP, changes in plasma EV-derived IL-1β, TNF-α and IL-6 levels were significantly associated with changes in the severity of postural instability and gait disturbance (PIGD) and cognition. Baseline plasma EV-derived IL-1β, TNF-α, IL-6, and IL-10 levels were significantly associated with the severity of PIGD and cognitive symptoms at follow-up, and PwP with elevated IL-1β and IL-6 levels exhibited significant progression of PIGD over the study period.

Conclusion: These results suggested the role of inflammation in PD progression. In addition, baseline levels of plasma EV-derived proinflammatory cytokines can be used to predict the progression of PIGD, the most severe motor symptom of PD. Additional studies with longer follow-up periods are necessary, and plasma EV-derived cytokines may serve as effective biomarkers of PD progression.

## INTRODUCTION

Parkinson’s disease (PD) is the second common neurodegenerative disease [1]. In addition to its well-known pathogeneses—such as α-synuclein accumulation, mitochondrial dysfunction, and proteasomal ubiquitin system abnormality—PD is characterized by the loss of dopaminergic neurons caused by systemic and local inflammation in the brain (neuroinflammation) [2]. Postmortem studies of PD have identified microglia and immune response activation [3–6], and epidemiological evidence strongly suggests that inflammatory bowel disease is associated with PD [7] and that nonsteroidal anti-inflammatory drugs have a risk-reduction effect on PD [8]. Moreover, studies have observed elevated levels of proinflammatory cytokines in cerebrospinal fluid and peripheral blood samples collected from people with PD (PwP) [9–13], supporting the link between PD and inflammation. However, the short half-life of free cytokines in peripheral blood and the considerable fluctuations in the abundance of cytokines due to environmental stress result in variabilities and inconsistencies in individuals’ blood cytokine profiles [14, 15]. Alternatively, cytokines derived from peripheral blood extracellular vesicles (EVs) can be used for the measurement of cytokine profiles [16]. EVs are tiny vesicles with lipid membranes and contain proteins, lipids, and nucleotides, which are responsible for cell-to-cell signal transmission. EVs maintain their integrity when crossing the blood–brain barrier (BBB) [17]. Through EV-derived cytokines, donor cells can modulate the inflammatory reactions of recipient cells. The profiles of cytokines derived from blood EVs may reflect systemic inflammation and have thus been utilized as biomarkers in some malignant and infectious diseases [18, 19].

Our research group previously conducted a cross-sectional study comparing the plasma EV-derived cytokine profiles in PwP with those in healthy controls (HCs); the results revealed that the plasma EV-derived IL-1β and TNF-α levels observed in the PwP were significantly higher than those observed in the HCs, and the severity of cognitive dysfunction in the PwP was significantly associated with IL-1β, IL-6, IL-10, and TNF-α levels [20]. We conducted the present follow-up study to further investigate the association between changes in plasma EV-derived cytokine profiles and the progression of clinical manifestations of PD and to evaluate the use of baseline plasma EV-derived cytokine profiles as biomarkers of the clinical progression of PD.

## MATERIALS AND METHODS

### Study participants

A total of 146 participants (101 PwP and 45 HCs) were enrolled in this study. The PD diagnoses were based on the UK Parkinson’s Disease Society Brain Bank Diagnostic Criteria [21]. Only individuals with mild to moderate PD, defined as stage I to III PD according to the Hoehn and Yahr scale, were included in the study. The HCs had no known neurodegenerative, psychiatric, or major systemic (malignant neoplasm and chronic kidney disease) diseases and were regularly followed up at outpatient clinics for chronic conditions (hypertension, diabetes, or hyperlipidemia). This study was approved by the Joint Institutional Review Board of Taipei Medical University (approval no. N201609017 and N201801043).

### Clinical assessments

We interviewed all the PwP and HCs to obtain their baseline demographic data. The cognitive function of all participants was evaluated by trained nurses using the Taiwanese versions of the Mini-Mental State Examination (MMSE) and Montreal Cognitive Assessment (MoCA). Dementia was defined as an MMSE score of <26. All PwP were evaluated using Parts I, II, and III of the Unified Parkinson’s Disease Rating Scale (UPDRS) during an outpatient visit. The duration between the most recent dose of anti-PD medication and completion of Part IV of the UPDRS was not recorded; the PwP were assumed to be on their prescribed medications. Tremor, akinetic rigidity (AR), and postural instability and gait disturbance (PIGD) subscores for each patient were calculated using the corresponding subitems on Part III of the UPDRS (subitems 20 and 21 for tremor subscores; subitems 27–30 for the PIGD subscores; and subitems 18, 22, 23, 24, 25, 26, and 31 for the AR subscores) according to a modified version of a method described in a previous study [22].

### Plasma EV isolation and validation

In the plasma EV isolation process, venous blood samples were collected from all participants. Whole blood was centrifuged at 13,000 × g for 20 minutes to isolate the plasma. Subsequently, an exoEasy Maxi Kit was used to isolate the exosomes from 1 mL of plasma collected from each participant, in accordance with the manufacturer’s instructions. Finally, the exosomes were eluted from the column. Approximately 400 μL of elution solution was obtained from each sample. The isolated EVs were validated through. (1) EV markers, including the presence of CD63, CD9, tumor susceptibility gene 101 protein and negative of cytochrome c. (2) The nanoparticle tracking analysis, which demonstrated the majority of the size of EV is within 50–100 nm. (3) The morphology from the electron microscopy analysis. The validation method employed in the present study is described in the literature [23, 24].

### Western blot analysis of plasma EV-derived cytokines

The isolated plasma EVs were directly lysed using protein sample buffer (RIPA Lysis Buffer, Millipore) and analyzed using protein-sodium dodecyl sulfate and polyacrylamide gels. Antibodies against IL-1β (Cell Signaling Technology, Cat. #12242, 1:1000), IL-6 (Cell Signaling Technology, Cat. #12912, 1:1000), TNF-α (Cell Signaling Technology, Cat. #11948, 1:1000), TGF-β (Abcam, Cat. ab215715, 1:1000), and IL-10 (Abcam, Cat. ab133575, 1:1000) were used. The antibodies were diluted in tris-buffered saline containing 0.1% Tween 20 and 5% bovine serum albumin. Secondary antibodies, namely horseradish peroxidase (HRP)-conjugated antimouse IgG (115-035-003) and HRP-conjugated antirabbit IgG (111-035-003), were purchased from Jackson ImmunoResearch (West Grove, PA, USA). The protein blot intensities were quantified using Image J software. The cytokine expression levels were normalized to the heat-shock protein expression levels 70 (Proteintech, Cat. 10995-1-AP, 1:2000). To ensure that the data from different gels could be compared, all the data from each gel were normalized to the control group average for the same gel.

### Statistical analysis

All statistical analyses were performed using SPSS for Windows 10 (version 19; SPSS, Chicago, IL, USA). A chi-square test was used to compare the gender distributions of the PwP and HCs. A linear mixed model was used to test whether the changes in the plasma EV-derived cytokine profiles of the PwP and HCs differed after adjustment for age and sex. Generalized linear model was used to investigate the association between the changes in the plasma EV-derived cytokine levels and changes in the clinical parameters of the PwP after adjustment for age, sex, and disease duration. Multivariable logistic regression with adjustment for age, sex, and disease duration was used to investigate the associations between the plasma EV-derived cytokine profiles and clinical parameters of the PwP at follow-up. A repeated-measures analysis of variance with estimated marginal means was used to compare the changes in PIGD subscores from baseline to follow-up between the PwP with and without elevated baseline IL-1β and IL-6 levels (upper one-third). A *p* value of <0.05 was considered statistically significant.

### Consent for publication

All authors have read and approved the final version of the manuscript. All authors agree to the present state of authorship and have signed a statement attesting to authorship.

### Availability of data and materials

Please contact the corresponding author (CT Hong). Access to data and materials requires permission from the TMU-JIRB.

## RESULTS

**Figure 1:**
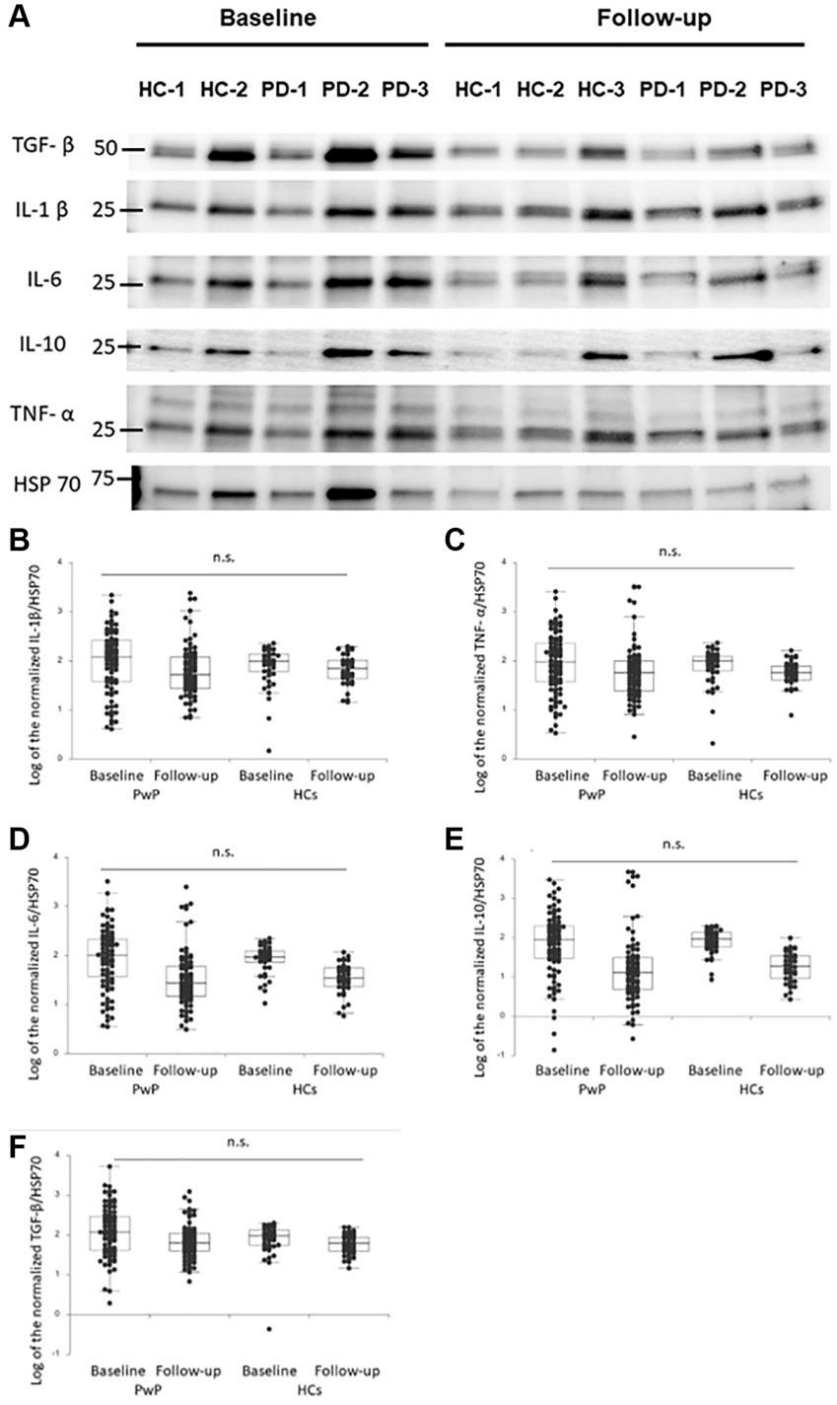
(**A**) Baseline and follow-up plasma extracellular vesicle (EV)-derived cytokine profiles of people with Parkinson’s disease (PwP) and healthy controls (HCs). Representative protein blot images of cytokines: interleukin (IL)-1β, IL-6, IL-10, tumor necrosis factor (TNF)-α, and transforming growth factor (TGF)-β. Heat-shock protein 70 was the protein loading control. (**B**–**F**) Comparison of plasma EV-derived IL-1β (**B**), TNF-α (**C**), IL-6 (**D**), IL-10 (**E**), and TGF-β (**F**) levels of PwP and HCs at baseline and follow-up. Data are presented as a dot plot with median values and first and third quartiles. Abbreviation: n.s.: nonsignificant.

The demographic data collected at baseline and at the 1-year follow-up are presented in Table 1. A total of 101 PwP and 45 HCs were followed up. The changes in the plasma EV-derived cytokine levels of the PwP and HCs did not differ significantly. We observed no significant changes in the plasma EV-derived cytokine levels of the PwP and HCs from baseline to follow-up after adjustment for age and sex (Figure 1A, representative image, and Figure 1B–1F, dot plot for the comparison).

**Table 1:** Demographic data of study participants.

	**HCs, *n* = 45**	**PwP, *n* = 101**
Age (years)	65.57 ± 10.6	68.87 ± 7.76
Women (%)	16	48
Baseline		
MMSE	28.22 ± 1.55	25.28 ± 4.15
MoCA	23.84 ± 3.15	20.83 ± 5.70
Disease duration	−	2.65 ± 2.24
UPDRS-II	−	8.49 ± 5.58
UPDRS-III	−	22.79 ± 9.30
1-year follow-up		
MMSE	26.61 ± 4.08	24.87 ± 5.61
MoCA	23.34 ± 5.64	20.79 ± 6.45
UPDRS-II	−	11.26 ± 6.41
UPDRS-III	−	21.24 ± 9.39

Abbreviations: HC: healthy control; PwP: people with Parkinson’s disease; MMSE: Mini-Mental State Examination; MoCA: Montreal cognitive assessment; UPDRS: unified Parkinson’s disease rating scale.

We investigated the associations between changes in plasma EV-derived cytokine profiles and changes in clinical parameters among the PwP (Table 2). Changes in the PIGD subscore of the UPDRS-III were significantly associated with changes in plasma EV-derived IL-1β (B = 232.948, *p* = 0.001), TNF-α (B = 231.173, *p* = 0.050), and IL-6 (B = 301.64, *p* < 0.001) levels after adjustment for age, sex, and disease duration. Changes in the total scores on MMSE were significantly negatively associated with plasma EV-derived IL-1β (B = −27.828, *p* = 0.001), TNF-α (B = −34.778, *p* = 0.032), and IL-6 (B = −34.294, *p* = 0.003) levels after adjustment for age, sex, and disease duration. Changes in the total scores on MoCA were significantly negatively associated with plasma EV-derived IL-1β (B = −92.673, *p* = 0.002), TNF-α (B = −30.596, *p* = 0.012), IL-6 (B = −28.393, *p* = 0.001) and IL-10 (B = −35.454, *p* = 0.049) levels after adjustment for age, sex, and disease duration.

**Table 2:** Generalized linear model for investigating the association between the change of plasma EV cytokines with the change of clinical severity (as dependent variable) in people with Parkinson’s disease, presented as B value (*p* value) after adjusting age, sex and disease duration.

	UPDRSII ^*^Follow	UPDRSIII ^*^Follow	Tremor ^*^Follow	AR ^*^Follow	PIGD ^*^Follow	MMSE ^*^Follow	MoCA ^*^Follow
IL-1β	1.891 (0.825)	2.269 (0.675)	−70.221 (0.691)	−23.922 (0.931)	**232.948 (0.004)**	**−27.828 (0.001)**	**−92.673 (0.002)**
TNF-α	−4.004 (0.743)	−0.848 (0.912)	−68.001 (0.789)	−103.14 (0.517)	**231.173 (0.050)**	**−34.778 (0.032)**	**−30.596 (0.012)**
IL-6	6.210 (0.482)	8.088 (0.143)	−111.77 (0.543)	107.236 (0.352)	**301.64 (<0.001)**	**−34.294 (0.003)**	**−28.393 (0.001)**
IL-10	−15.130 (0.395)	−12.361 (0.271)	−193.14 (0.604)	−361.08 (0.122)	182.86 (0.294)	−43.928 (0.067)	**−35.454 (0.049)**
TGF-β	**−28.210 (0.012)**	−1.772 (0.807)	272.863 (0.237)	−94.099 (0.530)	−5.969 (0.957)	17.797 (0.237)	14.164 (0.209)

Abbreviations: UPDRS: unified Parkinson Disease rating scale; AR: akinetic rigidity; PIGD: postural instability and gait disturbance; MMSE: mini-mental status examination; MoCA: Montreal cognitive assessment.

**Figure 2:**
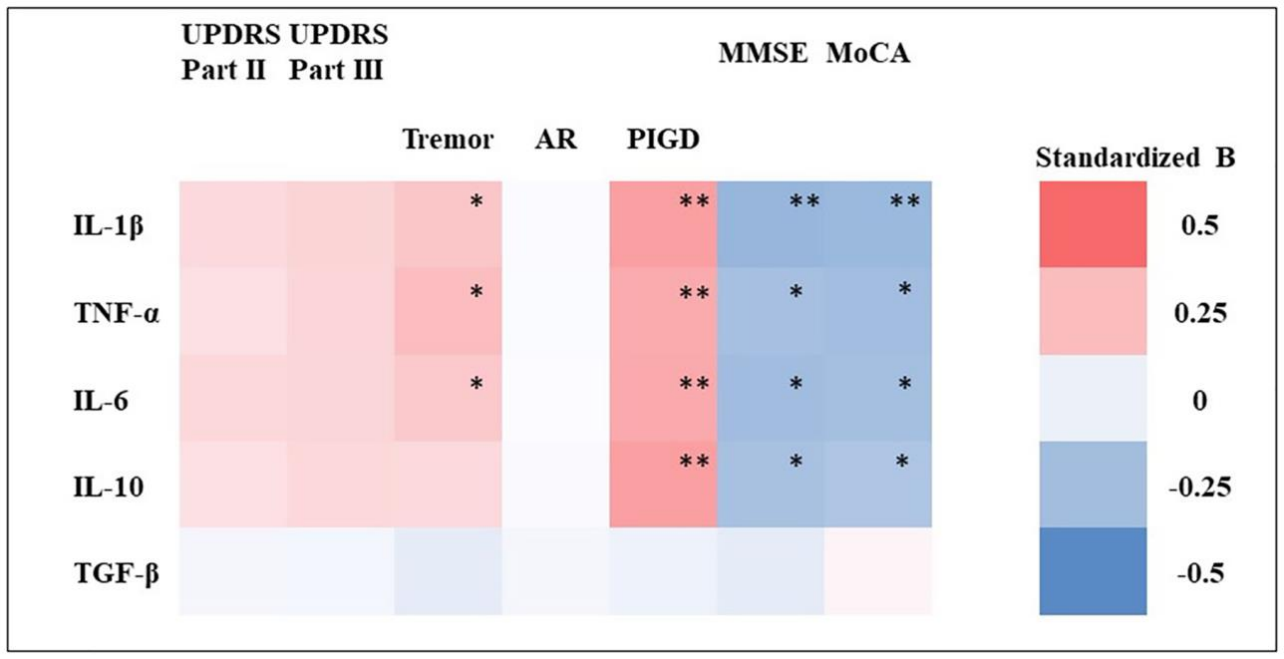
**Heatmap of association between baseline plasma EV-derived cytokine levels and clinical parameters of PwP at follow-up.** A logistic regression model was used to investigate the association between baseline plasma EV-derived cytokine (IL-1β, IL-6, IL-10, TNF-α, and TGF-β) levels and motor symptoms (as assessed using their scores on Parts II and III of the UPDRS and their tremor, akinetic rigidity [AR], and postural instability and gait disturbance [PIGD] subscores) as well as cognitive function (as assessed using their MMSE and MoCA scores). The association is presented in terms of standardized β values. The detailed results of the regression are presented in Supplementary Table 2. ^*^p < 0.05, ^**^p < 0.01.

We further investigated the association of PD clinical parameters at follow-up with baseline plasma EV-derived cytokine levels. After adjustment for age, sex, and disease duration, we observed that plasma EV-derived IL-1β, TNF-α, IL-6, and IL-10 levels were not significantly associated with the scores on Parts II and III of the UPDRS at follow-up (Figure 2 and detail in Supplementary Table 1). However, after dividing the scores on Part III of the UPDRS into tremor, AR, and PIGD subscores, we noted that baseline plasma EV-derived IL-1β, TNF-α, and IL-6 levels were significantly and positively associated with tremor subscores at follow-up and that baseline plasma EV-derived IL-1β, TNF-α, IL-6, and IL-10 levels were significantly and positively associated with PIGD subscores at follow-up. Regarding the cognitive symptoms of PD, plasma EV-derived IL-1β, TNF-α, IL-6, and IL-10 levels were significantly and negatively associated with MMSE and MoCA scores at follow-up.

**Figure 3:**
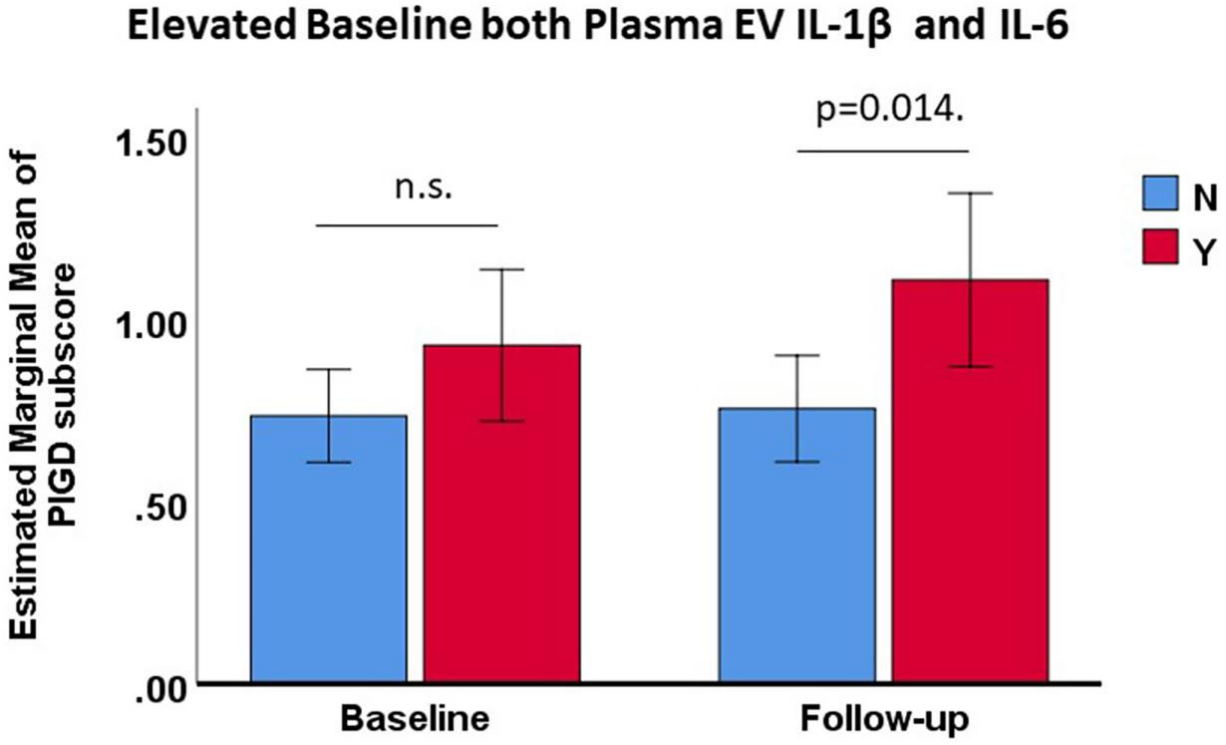
**Changes in estimated marginal mean PIGD subscores of PwP with and without elevated baseline plasma EV-derived IL-1β and IL-6 levels (upper one-third) at baseline and follow-up, adjusted for age, sex, and disease duration.** Data are presented as mean values with 95% confidence intervals. Abbreviation: n.s.: nonsignificant.

Finally, we grouped the PwP on the basis of their baseline plasma EV-derived cytokine levels; the cutoff point was defined as the upper one-third of baseline plasma EV-derived cytokine level. The PwP with elevated baseline plasma EV-derived IL-1β and IL-6 levels generally had higher baseline PIGD subscores than did those with lower two-third plasma EV-derived IL-1β and IL-6 levels, and the difference between these subgroups increased and became significant at follow-up after adjustment for age, sex, and disease duration (Supplementary Table 2). The baseline PIGD subscore of the PwP with elevated plasma EV-derived IL-1β and IL-6 (*n* = 29) after adjustment for age, sex, and disease duration was higher than that of the remaining PwP by 0.194 points (95% CI: 0.439–0.052, *p* = 0.12). At follow-up, the estimated marginal mean of PIGD subscore of the PwP with elevated plasma EV-derived IL-1β and IL-6 (*n* = 29) after adjustment for age, sex, and disease duration was higher than that of the remaining PwP by 0.353 points (95% CI = 0.633–0.072, *p* = 0.014; Figure 3). Therefore, the PwP with elevated IL-1β and IL-6 levels tended to exhibit poorer cognition (as assessed using their MMSE and MoCA scores) than did those without elevated IL-1β and IL-6 levels at both baseline and follow-up (Supplementary Table 2).

## DISCUSSION

This study demonstrated that changes in plasma EV-derived cytokine profiles were associated with the clinical progression of PD. In addition, baseline plasma EV-derived cytokine levels were associated with clinical outcomes, including tremor and PIGD subscores and cognition, at follow-up. PwP with elevated baseline levels of plasma EV-derived proinflammatory cytokines, namely IL-1β and IL-6, exhibited significant PIGD progression (as assessed using the changes in their PIGD subscores) and had poorer cognition than did those without elevated IL-1β and IL-6 levels at both baseline and follow-up. These results indicate that plasma EV-derived cytokine profiles may serve as effective biomarkers of PD and reflect the key role of inflammation in the severity and progression of PD.

Plasma EVs are among the most promising sources of PD biomarkers [25]. EV-derived α-synuclein has remained the most frequently studied target cytokine, with substantial evidence supporting its applicability in distinguishing among HCs and patients with typical and atypical PD [26–30]. EV-derived tau, β-amyloid, neurofilament light chain, brain-derived neurotrophic factor, and insulin receptor substrate proteins have also been investigated in PD [23, 24, 31–33]. These results indicate the potential utility of EV-derived biomarkers in the prognosis of PD. Considering the major role of inflammation in PD, researchers are continuing to study the use of blood cytokine profiles as PD biomarkers. However, the results of these studies have remained inconclusive. A meta-analysis involving 2,654 participants revealed that the blood levels of several proinflammatory cytokines in PwP are significantly higher than those in HCs, but high heterogeneity levels were observed among the included studies [34]. In another study, researchers evaluated the use of blood cytokine profiles in the prediction of PD progression and discovered that anti-inflammatory profiles were associated with slower motor progression [35]. Another study revealed that the copresence of elevated blood TNF-α/IL-10 levels and mild cognitive impairment could predict early conversion of idiopathic rapid-eye-movement sleep behavior disorders into neurodegenerative synucleinopathies [36]. The inconsistent results may result from the highly fluctuated blood cytokines level upon different physical conditions and the short half-life of blood-free-form cytokines. Therefore, scholars move forward to establish the utility of blood EV-derived biomarkers in PD; moreover, because the stability of EVs is hypothesized for the researchers to avoid the imprecision associated with fluctuations in free cytokine levels due to transient surges or degradation, the use of EV-derived biomarkers may more precisely reflect the severity of systemic inflammation. In addition, due to the BBB crossing capability, a portion of blood EVs are brain-originated, and the plasma EV cytokine may also reflect the condition of neuroinflammation. Therefore, in the present study, we expanded upon our previous research by investigating the association between changes in plasma EV-derived cytokine profiles and the progression of PD. In the present study, we discovered that changes in plasma EV-derived cytokine levels were significantly associated with changes in total scores on PIGD subscore of the UPDRS-III, MMSE and MoCA, indicating that plasma EV-derived cytokines may serve as indicators of PD progression. Cognitive impairment is a crucial nonmotor symptom of PD, and the development of PD dementia causes substantial functional disability in PwP [37, 38], thereby limiting the options for PD management. Deep brain stimulation, a potent surgical treatment for PD, is not indicated for PwP who have dementia [39]. The detection of cognitive impairment in PwP requires a series of time-consuming neuropsychological tests [40]. Identifying the association between the changes in plasma EV synaptic protein levels and the changes in MoCA scores can be an alternative to detect cognitive impairments in PwP through peripheral blood examinations.

Furthermore, we identified a significant association between baseline plasma EV-derived cytokine levels and follow-up PIGD subscores. Compared with the relatively benign course of the TD subtype of PD, PIGD is associated with a greater brain pathological burden; rapid progression; and higher risks of dementia, falls, and mortality [41–44]. Several studies have revealed associations between blood levels of inflammatory markers and the progression of PD. For example, a study identified elevated blood IL-6 as an independent risk factor for mortality [45]. Another study revealed that blood IL-12p40 levels could be used to distinguish between a cohort of PwP with “poor motor (function) with cognitive impairment” and that of those with “benign motor (function)” [46]. Furthermore, blood IL-8, monocyte chemotactic protein 1, and macrophage inflammatory protein 1-β levels can be used to predict disease progression in PwP with variants of the leucine-rich repeat kinase 2 gene [47]. In the present study, follow-up PIGD subscores were positively associated with baseline plasma EV-derived cytokine levels, which reflects the major role of inflammation in the progression of PD.

The main strength of the present study is that it is the first to investigate changes in plasma EV-derived cytokine profiles and the association of changes in plasma EV-derived cytokine levels with disease progression in a PD cohort. The results of this study may serve as a reference for future research on the role of inflammation in the progression of PD. The significant association we identified between plasma EV-derived cytokine profiles with PIGD subscore, MMSE and MoCAs reflect the relationship between systemic inflammation and neurodegeneration. PIGD is the most detrimental motor symptom of PD, and at present, there is no good predictor of the progression of PIGD symptoms. Although no single cytokine can be used to accurately predict PD progression, cytokine panels analyzed using artificial neuronal networks have been effectively used to predict neurological outcomes after stroke in previous studies [48, 49]. In addition, the merit of the present study includes the association between the change of clinical symptoms with the plasma EV cytokine, which hints that these inflammatory biomarkers could serve as treatment response indicators. Considering the lack of available disease-modifying treatments for PD, the modulation of systemic inflammation is a practical therapeutic target, and plasma EV-derived cytokine profiles may serve as an effective indicator of patients’ therapeutic responses.

However, the present study has some limitations. The cytokine levels were measured using a semiquantitative Western blot analysis method, and the lack of absolute values limits the clinical applications of our findings. The small quantity of plasma EV-derived cytokines in the collected samples prevented us from performing a chemiluminescence assay; this is because chemiluminescence assay kits have not yet been optimized for ultrasensitive detection, despite the increasing frequency with which they are being applied in quantitative studies. In addition, the selection of HSP-70 as the control for the cytokine protein quantification of plasma EV is not undisputable. Two types of EV proteins are used for this role: membrane proteins and intravesicular proteins. Membrane proteins include CD9, CD63, and CD81; intravesicular proteins include TSG101, annexins, and chaperone proteins, such as HSP-70. Since cytokines are also intravesicular, we used the HSP-70 as the internal control. Currently, there is no clear evidence about the influence of PD pathology on the level of EV HSP-70, but it is a limitation of the present study. Second, the 1-year follow-up period was insufficient for tracking the progression of PD, especially its cognitive symptoms. Third, the motor assessment was performed while the PwP were on medications, and the severity of motor symptoms may have been masked by the effects of dopaminergic medications. Lastly, inflammation is a commonly shared pathogenesis of several neurological diseases, which is not specific to PD. Therefore, the HCs in the present study may be contaminated with concurrent neurological diseases, which accounts for the non-significant difference between PD and HCs. However, this approach also delineated the role of inflammation in the progression of PD, which helped to develop further anti-inflammation-based disease modification treatment of PD.

In conclusion, the present study revealed an association between changes in plasma EV-derived cytokine levels and the progression of motor and cognitive symptoms in PwP. Baseline plasma EV-derived cytokine levels were also associated with clinical outcomes, and elevated plasma EV-derived IL-1β and IL-6 levels were identified as predictors of the progression of PIGD. These findings indicate that plasma EV-derived cytokines may serve as effective biomarkers of PD progression and reflect the key role of inflammation in the pathogenesis of PD. Additional studies with longer follow-up periods are necessary to further evaluate the predictive and prognostic value of plasma EV-derived cytokine profiles.

## Supplementary Materials

Supplementary Tables
