# Supplementary material for: Cytokine profiles of plasma extracellular vesicles as progression biomarkers in Parkinson’s disease
Source: Aging (Albany NY). 2023 Mar 9;15(5):1603–14. doi: 10.18632/aging.204575 (PMC10042681; doi:10.18632/aging.204575)
Supplement: Supplementary Tables [file aging-15-204575-s001.pdf]

SUPPLEMENTARY TABLES

Supplementary Table 1. Association between the baseline plasma EV cytokines with the change of clinical severity in people with Parkinson's disease with the adjustment of age, sex and disease duration, presented as standardized B and *p* value.

	UPDRSII	UPDRSIII	Tremor	AR	PIGD	MMSE	MoCA
IL-1β	0.160 (0.125)	0.178 (0.089)	**0.220 (0.033)**	0.051 (0.634)	**0.337 (0.001)**	**−0.288 (0.003)**	**−0.276 (0.005)**
TNF-α	0.137 (0.198)	0.174 (0.102)	**0.247 (0.018)**	0.049 (0.653)	**0.302 (0.003)**	**−0.235 (0.019)**	**−0.252 (0.011)**
IL-6	0.164 (0.120)	0.170 (0.107)	**0.213 (0.041)**	0.056 (0.603)	**0.306 (0.002)**	**−0.256 (0.010)**	**−0.242 (0.014)**
IL-10	0.139 (0.193)	0.163 (0.125)	0.160 (0.130)	0.048 (0.660)	**0.334 (0.001)**	**−0.231 (0.021)**	**−0.210 (0.036)**
TGF-β	0.027 (0.802)	0.026 (0.812)	–0.020 (0.850)	0.028 (0.796)	0.007 (0.946)	−0.024 (0.812)	0.080 (0.431)

Abbreviations: UPDRS: unified Parkinson Disease rating scale; AR: akinetic rigidity; PIGD: postural instability and gait disturbance; MMSE: mini-mental status examination; MoCA: Montreal cognitive assessment.

Supplementary Table 2. The progression of clinical severity in people with Parkinson's disease with and without elevated baseline plasma extracellular vesicle (EV) interleukin (IL)-1β, IL-6 or both.

Plasma EV		IL-1β				IL-6				Both IL-1β&IL-6			
		L, *n* = 66	H, *n* = 35	*p*-value	*p* for trend	L, *n* = 66	H, *n* = 35	*p*-value	*p* for trend	L, *n* = 72	H, *n* = 29	*p*-value	*p* for trend
UPDRS-II	Baseline	8.09 ± 4.77	9.23 ± 6.88	0.332	**<0.001**	8.09 ± 4.90	9.23 ± 6.70	0.332	**<0.001**	8.33 ± 4.91	8.86 ± 7.06	0.669	**<0.001**
	Follow-up	10.36 ± 5.41	12.43 ± 7.92	0.183		10.88 ± 5.40	11.97 ± 8.03	0.418		10.94 ± 5.31	12.03 ± 8.58	0.442	
UPDRS-III	Baseline	22.65 ± 8.20	23.06 ± 11.21	0.836	0.162	22.80 ± 8.12	22.77 ± 11.32	0.987	0.067	22.79 ± 8.00	22.79 ± 12.10	0.999	0.125
	Follow-up	20.21 ± 7.63	23.17 ± 11.91	0.132		21.23 ± 8.19	21.26 ± 11.45	0.988		20.97 ± 8.12	21.90 ± 12.12	0.657	
Tremor	Baseline	0.38 ± 0.39	0.34 ± 0.27	0.518	0.124	0.39 ± 0.39	0.32 ± 0.29	0.327	0.124	0.38 ± 0.38	0.33 ± 0.28	0.520	0.200
	Follow-up	0.27 ± 0.29	0.32 ± 0.24	0.387		0.28 ± 0.30	0.31 ± 0.22	0.664		0.27 ± 0.29	0.33 ± 0.23	0.351	
AR	Baseline	1.06 ± 0.42	1.06 ± 0.52	0.970	0.209	1.07 ± 0.42	1.04 ± 0.52	0.769	0.079	1.07 ± 0.41	1.04 ± 0.56	0.732	0.132
	Follow-up	0.97 ± 0.38	1.16 ± 0.57	0.335		1.02 ± 0.41	0.95 ± 0.52	0.483		1.00 ± 0.41	0.98 ± 0.55	0.766	
PIGD	Baseline	0.71 ± 0.41	0.89 ± 0.77	0.128	0.104	0.70 ± 0.40	0.91 ± 0.78	0.071	0.175	0.70 ± 0.40	0.91 ± 0.84	0.118	0.123
	Follow-up	0.72 ± 0.48	1.05 ± 0.87	**0.016**		0.74 ± 0.48	1.01 ± 0.88	**0.046**		0.75 ± 0.47	1.05 ± 0.94	**0.035**	
MMSE	Baseline	26.03 ± 3.54	23.86 ± 4.86	**0.012**	0.382	25.74 ± 3.71	24.40 ± 4.81	0.123	0.482	25.63 ± 3.91	24.41 ± 4.67	0.186	0.401
	Follow-up	25.62 ± 5.39	23.46 ± 5.83	0.065		25.15 ± 5.62	24.34 ± 5.64	0.494		25.22 ± 5.50	24.00 ± 5.91	0.325	
MoCA	Baseline	21.72 ± 4.85	19.06 ± 6.78	**0.025**	0.794	21.14 ± 5.23	20.14 ± 6.46	0.409	0.848	21.10 ± 5.23	20.03 ± 6.79	0.401	0.918
	Follow-up	21.95 ± 5.47	18.63 ± 7.59	**0.013**		20.97 ± 6.35	20.45 ± 6.71	0.707		21.15 ± 6.22	19.90 ± 7.02	0.379	

Abbreviations: UPDRS: unified Parkinson Disease rating scale; AR: akinetic rigidity; PIGD: postural instability and gait disturbance; MMSE: mini-mental status examination; MoCA: Montreal cognitive assessment.
